# Alternatives to Acellular Dermal Matrix: Utilization of a Gore DualMesh Sling as a Cost-Conscious Adjunct for Breast Reconstruction

**Published:** 2017-02-10

**Authors:** Jacob N. Grow, James Butterworth, Paul Petty

**Affiliations:** ^a^Department of Plastic Surgery, University of Kansas Medical Center, Kansas City, Mo; ^b^Division of Plastic and Reconstructive Surgery, Mayo Clinic, Rochester, Minn

**Keywords:** breast reconstruction, acellular dermal matrix, mesh, tissue expansion, cost

## Abstract

**Objective**: This study seeks an alternative to acellular dermal matrix in 2-staged breast reconstruction while minimizing cost. It was hypothesized that use of a Gore DualMesh would allow for similar intraoperative tissue expander fill volumes, time to second-stage reconstruction, and number of postoperative fills compared with acellular dermal matrix at only a fraction of the expense. **Methods:** Retrospective analysis comparing Gore DualMesh (59 breasts, 34 patients), acellular dermal matrix (13 breasts, 8 patients), and total muscle coverage (25 breasts, 14 patients) for postmastectomy breast reconstruction was performed. Time to second-stage reconstruction, number of expansions, and relative initial fill volumes were compared between the 3 groups. Secondarily, complication rates were also considered, including seroma, infection, expander/implant explantation, removal of mesh, and capsular contracture. Statistical analysis was performed utilizing the Fisher exact test and the χ^2^ test for categorical variables and the Mann-Whitney *U* test for continuous variables. **Results:** Relative initial fill volumes, number of expansions, and time to second-stage reconstruction showed no statistical difference between the acellular dermal matrix and Gore DualMesh groups (*P* = .494, *P* = .146, and *P* = .539, respectively). Furthermore, the Gore DualMesh group underwent significantly fewer fills (*P* < .001) and had a higher relative initial fill volume (*P* < .001) than the total muscle coverage group. The additional cost per breast as a result of including DualMesh was on average $385 versus $4287 for acellular dermal matrix. Complication rates were similar between all 3 groups without statistically significant differences. **Conclusions:** Gore DualMesh represents a safe alternative to acellular dermal matrix for breast reconstruction with similar aesthetic results in certain patients at a fraction of the cost.

The use of acellular dermal matrix (ADM) has become an increasingly utilized and accepted adjunct to reconstruction of the postmastectomy breast.^1^^-^8 Its utilization has resulted in decreased time to second-stage reconstruction, number of expansions necessary, and increased total expander volume at initial operation.^7^^-^11 Added benefits include the decrease in inflammation, resulting in less capsular contracture.^12^^-^20 Despite this, its utilization has sometimes gone uncovered by insurance plans and the subsequent expenses may therefore be cost-prohibitive to patients and surgeons alike.^21^^-^23 There are multiple breast reconstruction blogs and chat rooms that showcase individuals who are actively appealing the charges incurred with the use of ADM.^24^^,^^25^ This has prompted a look at alternative sources to reproduce the gains of ADM in 2-staged breast reconstruction while minimizing the cost. It was hypothesized that the use of Gore DualMesh would function similarly to ADM with respect to intraoperative tissue expander fill volumes, number of postoperative fills, and time to second-stage procedure at only a fraction of the cost.

## METHODS

After institutional review board approval, a retrospective study of a single surgeon's experience comparing utilization of Gore DualMesh (Gore, Flagstaff, Ariz), ADM (Alloderm; Lifecell, Bridgewater, NJ), and total muscle coverage (TMC) was undertaken. A single surgeon's experience over an 18-month period was chosen to best standardize for the surgical technique. Patients having undergone both first- and second-stage breast reconstructions during this time period were included for review. Patients who had prior reconstructions or augmentations were excluded, as was 1 patient who expired from unrelated health issues prior to second-stage reconstruction. Included in the review were 34 patients (59 breasts) undergoing reconstruction with Gore DualMesh, 8 patients (13 breasts) utilizing ADM, and 14 patients (25 breasts) having TMC. Common risk factors resulting in postoperative complications were compared between the groups, including diabetes mellitus, body mass index, smoking, and radiation therapy. As a secondary endpoint to address mesh safety, complications rates were considered across the course of reconstruction for both first- and second-stage procedures, which included seroma, infection, expander/implant explantation, removal of mesh, and capsular contracture. The primary objective was to determine the number of expansions, total expander volume filled at the first stage as a percentage of the final implant size, and time to second-stage reconstruction in each of the 3 groups. Statistical analysis was performed utilizing the Fisher exact test and the χ^2^ test for categorical variables and the Mann-Whitney *U* test for continuous variables. The analysis was performed comparing the outcomes of the Gore DualMesh arm with the ADM arm and the Gore DualMesh arm with the TMC arm. This review was not designed to compare outcomes between ADM and TMC, which has been previously investigated at length.

### Operative technique

**Figure 1:**
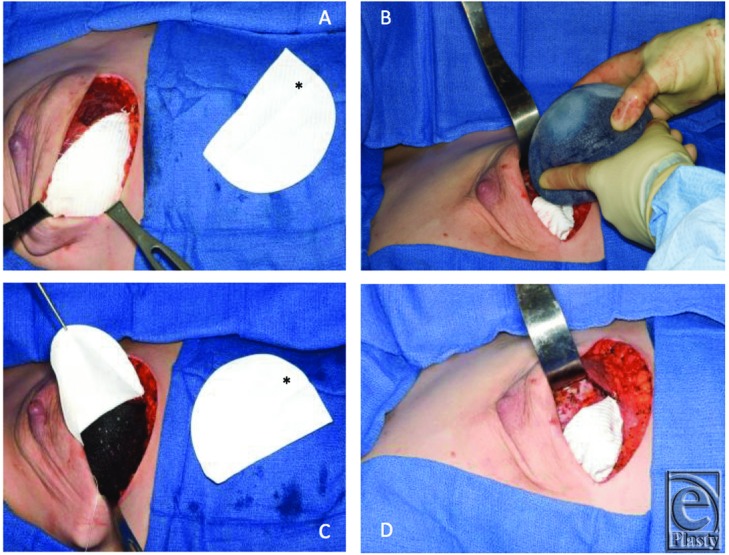
Operative technique for Gore-Tex DualMesh. (a) Dissected pocket prior to expander placement, with the mesh in the anticipated location (*Template). (b) Insertion of the expander. (c) Expander in the subpectoral position with the mesh retracted. (d) Final inset with the mesh sling at the inferior pole.

The operative techniques for TMC and ADM reconstructions have been well described and are therefore omitted from this article. The senior author's technique utilizing Gore DualMesh is similar to the reconstruction performed with ADM (Fig 1). The procedure begins with release of the serratus attachments at the lateral aspect of the breast pocket. A subpectoral plane is then developed superiorly to the upper pole and medially. This includes release of the inferior costal attachments of the pectoralis major to roughly the 9 o'clock position on the left breast and 3 o'clock on the right breast. A 10 × 15-cm piece of Gore DualMesh is typically utilized and may be cut in half in smaller breasted women, allowing for coverage of both breasts with a single piece. The mesh is cut to function as a lower-pole sling and shaped according to the curve of the inframammary fold. The mesh is then sewn to the inframammary fold. The expander is then placed into the pocket and conservatively filled with approximately 50 to 100 mL of normal saline. The free edge of the pectoralis muscle is then approximated to the free edge of the mesh, which is trimmed further for precise shape. The expander is then filled further to the tolerance of the muscle/mesh and skin to achieve tension-free closure without blanching of the mastectomy flaps. Two drains are placed, one in the superficial space and the other deep to the mesh. The drains are removed when the output is less than 30 mL per 24 hours for 2 consecutive days. The patients are maintained on antibiotics until removal of the drains.

**Figure 2:**
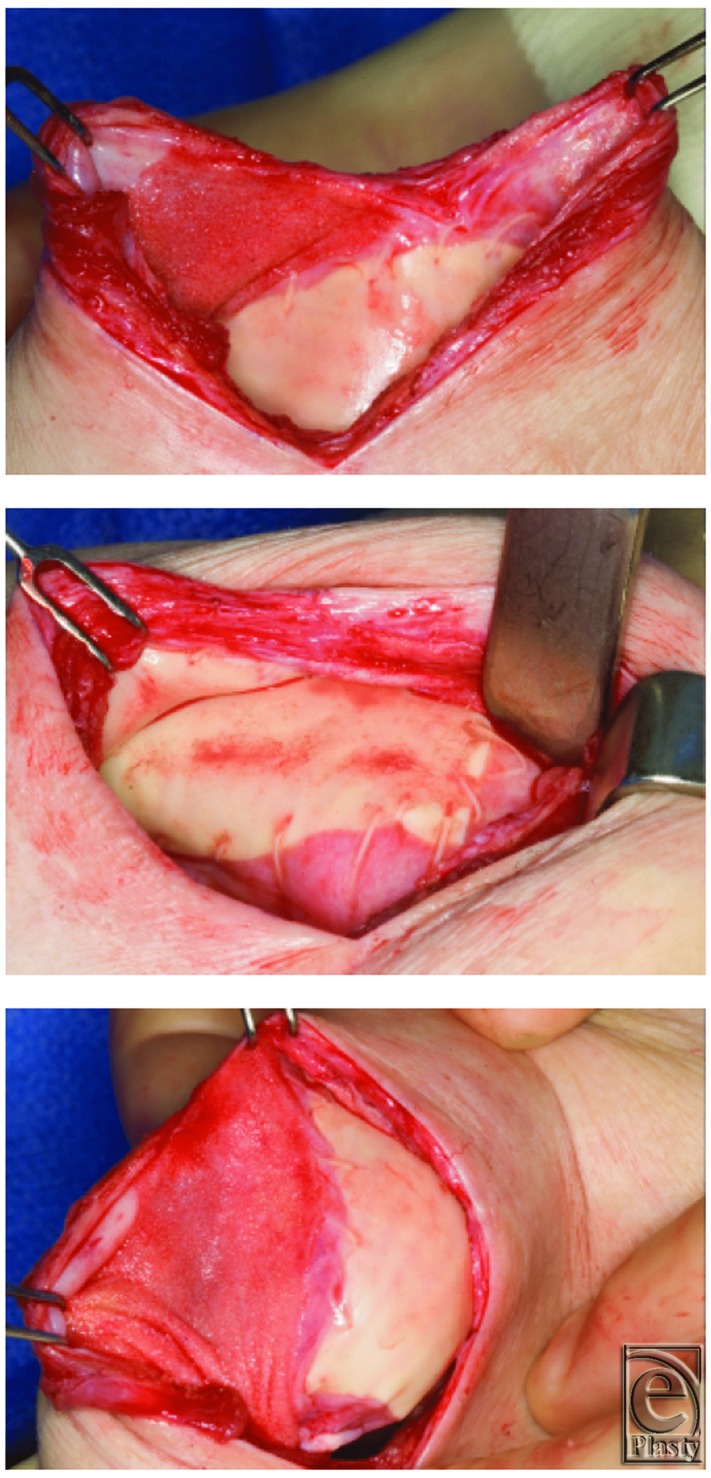
Gore-Tex at the second stage.

**Figure 3:**
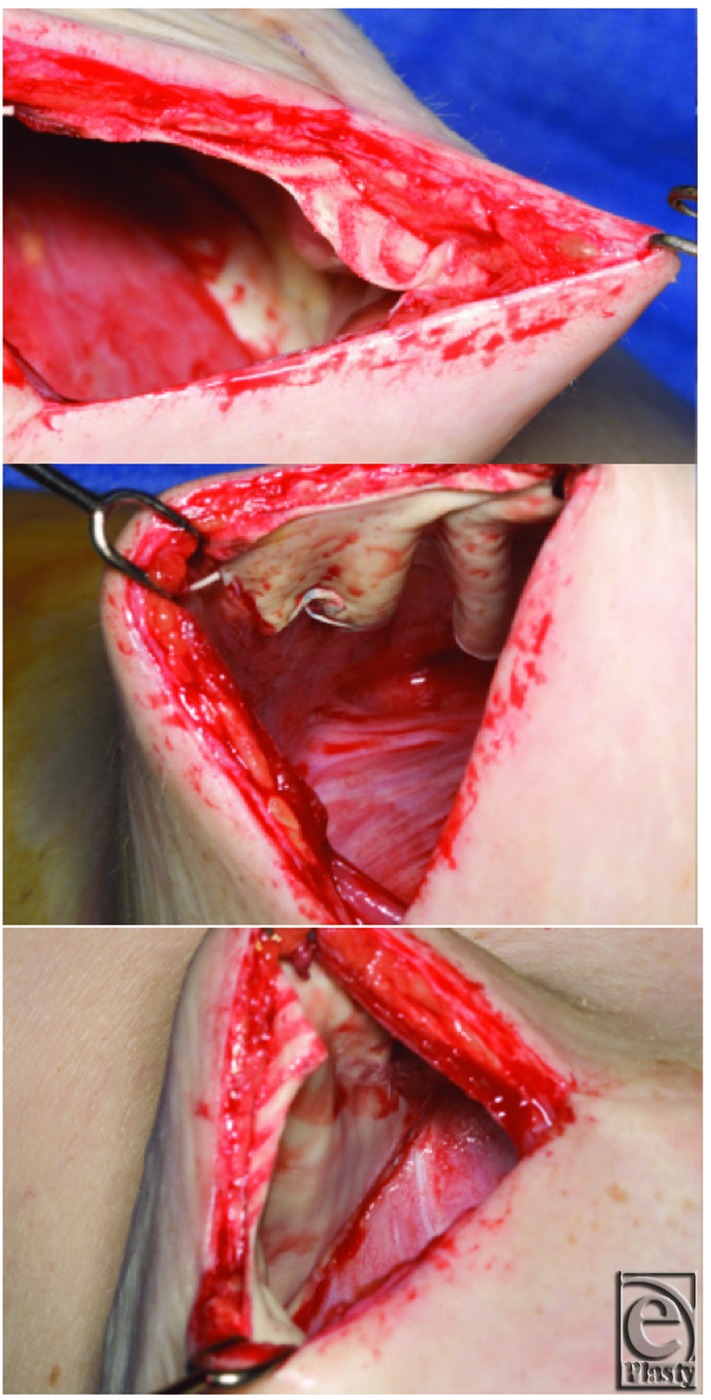
Poorly encapsulated Gore-Tex. Removed at the second stage.

At the second stage, the Gore DualMesh is generally encapsulated and left in place (Fig 2). If it is not encapsulated (Fig 3) or has resulted in a contour deformity, the DualMesh is easily removed without extending the operative time. In the setting of a contour deformity, the Gore-Tex is removed and the pocket modified with either release and suture or the addition of ADM to correct the deformity.

## RESULTS

All breasts (13/13) undergoing reconstruction with ADM underwent nipple- or skin-sparing mastectomies versus 58 of 59 breasts with Gore-Tex (*P* = 1). This is in contrast to the TMC group where 17 of 25 breasts underwent nipple- or skin-sparing mastectomies, which is a statistically significant difference when compared with the DualMesh group (*P* < .001) (Table 1). The average number of expansions between the first and second stages was 3.46 ± 2.02, 3.00 ± 3.22, and 5.7 ± 2.72 in the DualMesh, ADM, and TMC groups, respectively. These findings were similar between the ADM and DualMesh groups (*P* = .146) but statistically different between the TMC and DualMesh groups (*P* < .001). The average volume filled at the first stage as a percentage of the final implant volume was 81.9% ± 25.0%, 78.3% ± 26.7%, and 54.3% ± 13.1% in the Gore-Tex, ADM, and TMC groups, respectively. This was again similar between the DualMesh and ADM groups (*P* = .494) but statistically different between the TMC and Gore-Tex groups (*P* < .001). The average time to second-stage reconstruction was similar among the DualMesh, ADM, and TMC groups at 305.4 ± 97.4, 283.9 ± 84.2, and 372.5 ± 131.2 days, respectively (Table 2). There was no statistically significant difference between the ADM and Gore-Tex groups (*P* = .539) or the TMC and Gore-Tex groups (*P* = .165). The additional cost per breast using DualMesh was on average $385 versus $4287 for ADM (Alloderm; 8 × 16 cm).

**Figure 4:**
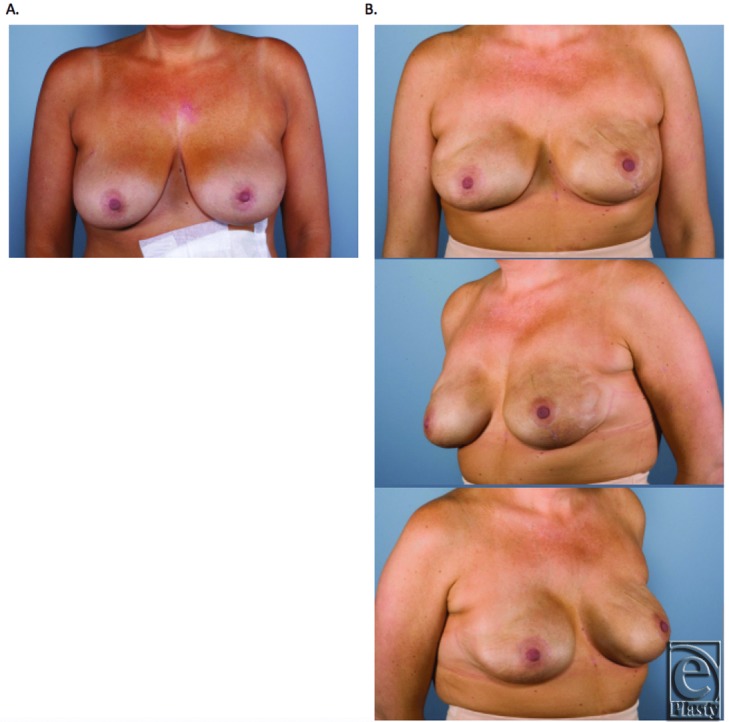
Capsular contracture with rippling. (a) Preoperation. (b) Postoperation.

**Figure 5:**
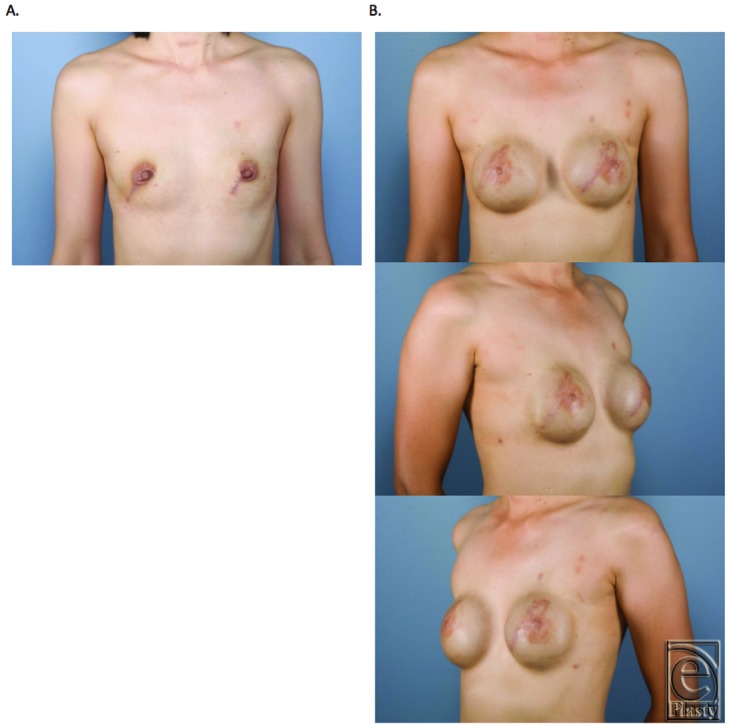
Capsular contracture. (a) Preoperation. (b) Postoperation.

The average follow-up from second-stage reconstruction was 14.8 months (range, 3.8-36 months). Risk factors were similar between all 3 groups without statistically significant differences (Table 1). All patients receiving radiation therapy developed capsular contracture regardless of reconstructive modality. This was based on the senior author's assessment using the Baker classification. Capsular contracture occurred in 7 of 59 breasts in the Gore DualMesh group (Table 3). Four breasts developed grade 2 contracture, and 3 breasts developed grade 3 contracture. Two of the breasts with capsular contracture also developed rippling (Fig 4). Within the breasts developing grade 3 contracture, 1 breast was irradiated and the other 2 were A cup size breasts in a thin patient (Fig 5). Within the breasts developing grade 2 contracture, one had developed a hematoma requiring evacuation after the second stage, one had a rupture prior to expansion, and one had rheumatoid arthritis on methotrexate therapy. The ADM group had 0 of 13 breasts with capsular contracture, a comparison that was not statistically significant (*P* = .337). Mesh removal took place at second stage in 10 of 59 breasts in the Gore DualMesh group. Four of these were replaced by ADM for correction of contour deformity, whereas 6 were simply removed and not replaced with any substitute. This compared with 0 of 13 breasts in the ADM group but again was not statistically significant (*P* = .191). Minor wound necrosis requiring no more than minor revision in the clinic was performed in 6 of 59 breasts in the Gore DualMesh group compared with 2 of 13 breasts in the ADM group (*P* = .626). Two breasts had cellulitis in the Gore DualMesh group compared with 1 in the ADM group (*P* = .467). Two breasts had expander explantation and replacement (one for leak and one for infection) in the Gore-Tex group compared with 1 breast in the ADM group (*P* = .467). Two breasts required hematoma evacuation in the DualMesh group and 1 in the ADM group (*P* = .467). No breasts in either group had seroma formation after drain removal or implant exchange.

## DISCUSSION

This study has obvious limitations in the sampled patient size and retrospective design. By pooling the data of the simple delayed mastectomies with the skin-/nipple-sparing mastectomies in the TMC group, a false elevation in the number of expansions and reduction in the amount of fill volumes at the first stage may occur. When removing these patients from the statistics, however, the significance is unchanged when compared with the Gore-Tex group in the number of expansions (*P* < .001), initial fill volume as a percentage of the final implant volume (*P* < .001), and time to second-stage reconstruction (*P* = .196). This suggests that utilizing a Gore DualMesh does indeed replicate the benefits of ADM in reducing the number of expansions and increasing the on-table fill volume. Although no statistical difference for time to second-stage procedure between the DualMesh and TMC cohorts was found, this result may be misleading. In the practice of the single surgeon whose patients were sampled in the study, time to secondary procedure was multifactorial and without standardization, regardless of expediency of reaching a final tissue expander volume. Factors such as operating room scheduling availability and patient preference influenced the timing of the second stage. There was no urgency for expander-to-implant exchange once filling was complete, as evidenced by the wide range of time to second-stage reconstruction between the 3 groups. This makes time to second-stage procedure a weaker endpoint than the total number of expansions required between the first and second stages as an indicator of efficiency.

**Figure 6:**
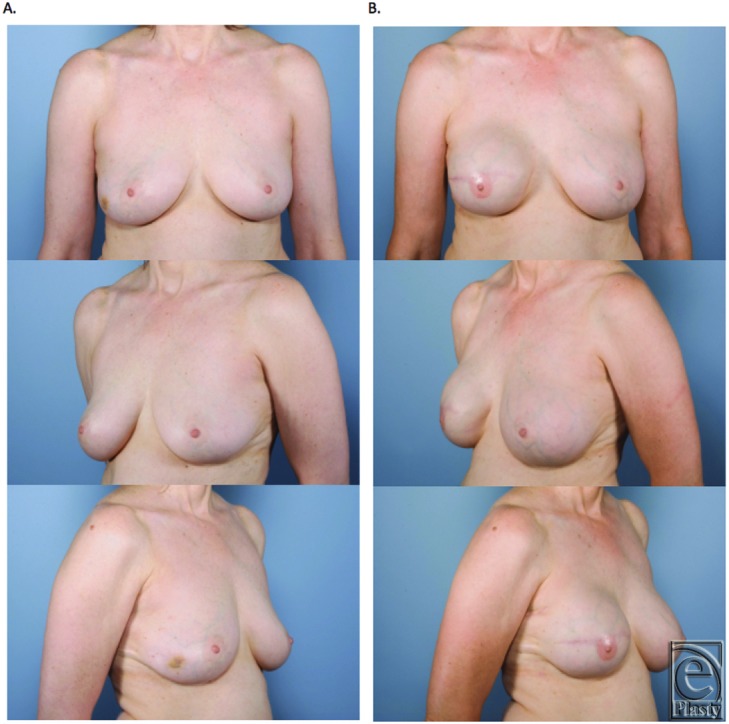
Unilateral skin-sparing mastectomy. Immediate reconstruction with Gore-Tex. (a) Preoperative. (b) Postoperation following expander-to-implant exchange.

**Figure 8:**
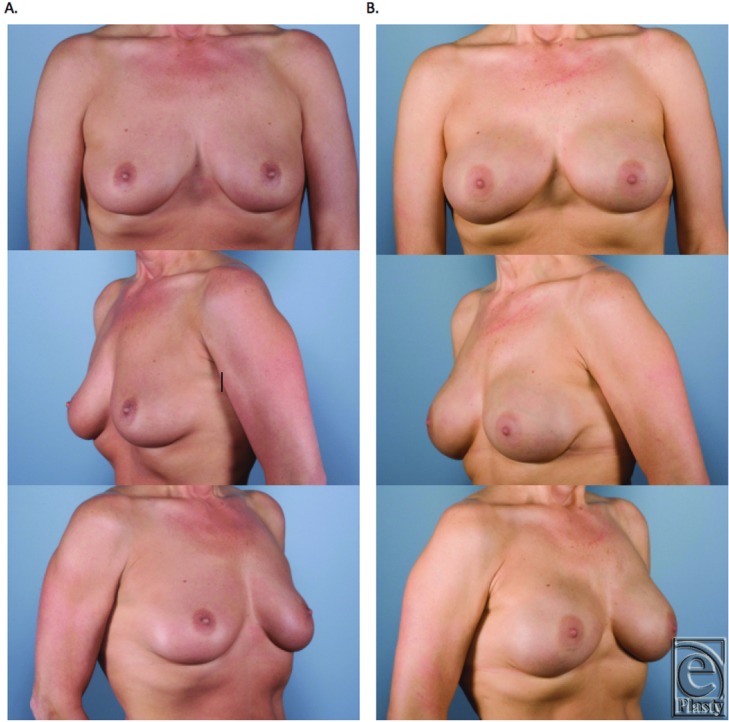
Bilateral nipple-sparing prophylactic mastectomies. Immediate reconstruction with Gore-Tex. (a) Preoperation. (b) Postoperation following expander-to-implant exchange.

Based on the surgeon's experience, the use of Gore DualMesh can achieve an excellent aesthetic outcome for patients (Figs 6 [Fig F7]-8). Those patients requiring radiation therapy and having either a thin body habitus or thin flaps (<8 mm) may experience rippling or capsular contracture and therefore should not undergo reconstruction with Gore-Tex in our experience. A short follow-up and a low power likely resulted in the statistically insignificant differences in the contracture rate, although a trend toward a higher incidence of contracture with DualMesh was evident. When capsular contracture did develop, most cases were minor Baker grade 2 and did not require revision. However, this complication certainly needs to be respected, as those who did undergo revision often required replacement of the Gore DualMesh with ADM, adding to the complexity and cost of the second stage.

One patient in this study underwent Gore-Tex reconstruction in one breast and ADM reconstruction in the other breast at the time of immediate bilateral breast reconstruction. This was due to the concern for future radiation therapy, with ADM placement on the oncologic side and DualMesh on the prophylactic side. However, no radiation therapy was required. Interestingly, the intraoperative fill volumes and the number of expansions were identical in both breasts. The patient suffered no complications and achieved a symmetric reconstruction without capsular contracture, bringing into question the relationship between DualMesh and capsular contracture. These findings may also support the theory that ADM prevents contracture, evidenced by the higher contracture rate in the Gore DualMesh group than in the ADM group.^12^^,^^13^^,^^15^^-^20

Although removal of the DualMesh occurred in 10 of the breasts at the second stage, this number may be misleading, as only 4 cases required removal out of necessity for contour deformities. The other 6 cases were electively explanted for lack of encapsulation. As a result, 6 of these cases were not true complications but for the purposes of transparency were reported as such. As Gore DualMesh is not a matrix, it does not incorporate and is easily peeled away from the surrounding tissue. Initially, Gore-Tex suture was used for insetting the mesh. After multiple patient complaints about painful, palpable knots requiring removal at the second stage, the Gore-Tex suture was replaced by absorbable PDS suture with resolution of symptoms. Aside from trends toward increased rates of capsular contracture following radiation therapy, the data do suggest the overall safety of utilizing DualMesh for reconstruction. Although future studies with a higher power will assist in identifying complication rates, our cohort confirms the early-phase proof of concept for safety and tolerance of the Gore-Tex.

On the basis of the results, we do not advocate that Gore DualMesh is a replacement for ADM, which has been shown to reduce the inflammatory response in the reconstructed breast.^14^^,^^20^^,^^26^ This is not a property shared by the DualMesh, which showed higher contracture rates than ADM. However, unlike the most commonly used ADM in breast reconstruction (Alloderm), Gore DualMesh is a sterile product and may not carry the increased risk of infection that has been reported with ADM.^27^^-^31 Further studies would be warranted to assess this risk profile.

Additional alternatives to ADM have recently been investigated as a means to temper reconstructive costs. Both the use of absorbable mesh and bioresorbable silk-based scaffold (SERI) for staged breast reconstruction show good aesthetic outcomes with acceptable complication rates at a fraction of the cost of ADM.^32^^-^34 Investigations modifying surgical technique to minimize the amount of ADM through a partial sling approach also represent a reasonable compromise to reduce material fees.^35^ Although the aforementioned studies lack the direct comparison with traditional ADM reconstruction that was considered in this study, they do appear to represent cost-effective alternatives to ADM reconstruction with similar results to Gore DualMesh.

The aim of this study was to demonstrate the proof of concept for a product with some of the benefits of ADM, a reasonable complication profile, and a significantly reduced cost. With current and upcoming changes to Medicare, the fate of ADM coverage in the setting of breast reconstruction is unknown. As mentioned previously, there are multiple examples of excessive hospital bills for ADM breast reconstruction where insurance companies consider its use “experimental” or unnecessary and therefore refused coverage. While Lifecell has a very charitable program to assist some of these women with the financial burden, if the refusal to cover ADM were to increase, the number of women requiring assistance may become too large. In addition, there are other ADMs on the market being used for breast reconstruction that may not have a benevolent backup program for the patient. As a result, alternative products are needed currently and may be even more important in the near future.

## Figures and Tables

**Figure 7 F7:**
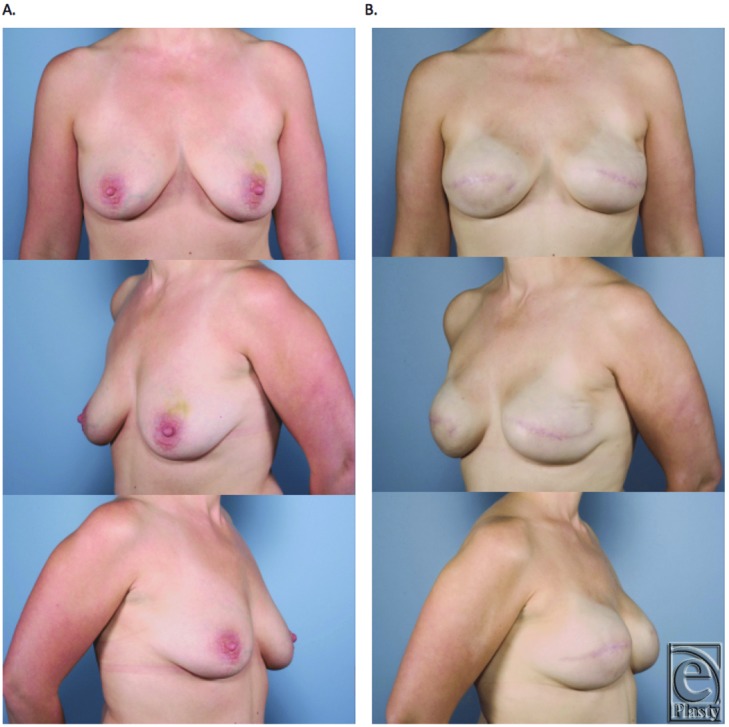
Bilateral skin-sparing mastectomies. Immediate reconstruction with Gore-Tex. (a) Preoperation. (b) Postoperation following expander-to-implant exchange.

**Table 1:** Patient characteristics*

	ADM (n =8 patients; 13 breasts), n (%)	Gore-Tex (n = 34 patients; 59 breasts), n (%)	TMC (n = 14 patients; 25 breasts), n (%)	ADM vs Gore-Tex/Gore-Tex vs TMC
Nipple-/skin-sparing	13/13 (100)	58/59 (98.3)	17/25 (68)	*P* = 1/*P* < .001
Diabetes mellitus	0/8 (0)	1/34 (2.9)	1/14 (7.1)	*P* = 1/*P* = .514
Radiation therapy	0/13 (0)	1/59 (1.7)	1/25 (4.0)	*P* = 1/*P* = .516
Smoking	0/8 (0)	2/34 (5.9)	2/14 (14.3)	*P* = 1/*P* = .578
Body mass index,	25.00 ± 4.85	24.78 ± 4.25	26.38 ± 5.88	*P* = .962/*P* = .425
mean ± SD				

*ADM indicates acellular dermal matrix; TMC, total muscle coverage.

**Table 2:** Outcomes*

	ADM(n =13 breasts)	Gore-Tex (n = 59 breasts)	TMC (n = 25 breasts)	ADM vs Gore-Tex/Gore-Tex vs TMC
Time to the second stage, mean ± SD, d	283.9 ± 84.2	305.4 ± 97.4	372.5 ± 131.2	*P* = .539/*P* = .165
Number of fills, mean ± SD	3.00 ± 3.22	3.46 ± 2.02	5.7 ± 2.72	*P* = .146/*P* < .001
% of final implant volume filled at the first stage, mean ± SD	78.3 ± 26.7	81.9 ± 25.0	54.3 ± 13.1	*P* = .494/*P* < .001

*ADM indicates acellular dermal matrix; TMC, total muscle coverage.

**Table 3:** Complications

	Acellular dermal matrix (n =13 breasts), n (%)	Gore-Tex (n = 59 breasts), n (%)	
Capsular contracture	0/13 (0)	7/59 (11.9)	*P* = .337
Mesh removal	0/13 (0)	10/59 (16.9)	*P* = .191
Infection/cellulitis	1/13 (7.7)	2/59 (3.4)	*P* = .467
Explantation	1/13 (7.7)	2/59 (3.4)	*P* = .467
Seroma	0/13 (0)	0/59 (0)	*P* = 1
Minor wound revision	2/13 (15.4)	6/59 (10.2)	*P* = .626
Hematoma	1/13 (7.7)	2/59 (3.4)	*P* = .467
